# Supplementary material for: Effects of information-induced behavioural changes during the COVID-19 lockdowns: the case of Italy
Source: R Soc Open Sci. 2020 Oct 7;7(10):201635. doi: 10.1098/rsos.201635 (PMC7657925; doi:10.1098/rsos.201635)
Supplement: Information index and other supplementary material [file rsos201635supp1.pdf]

Supplementary Materials
Effects of information–induced behavioral changes during the COVID–19 lockdowns: the case of Italy

Bruno Buonomo[1,*] and Rossella Della Marca[2]

[1] *Department of Mathematics and Applications, University of Naples Federico II, via Cintia, I-80126 Naples, Italy.*
buonomo@unina.it
[2] *Department of Mathematical, Physical and Computer Sciences, University of Parma, Parco Area delle Scienze 53/A, 43124 Parma, Italy.*
rossella.dellamarca@unipr.it

S.1 The information index

The information index is an important tool of behavioral epidemiology. In the seventies, V. Capasso proposed for the first time a behavioral–implicit SIR model, where the contact rate – until then taken as constant – was assumed to be a decreasing function of the infection prevalence I. In this way, the contact rate takes into account of the behavioral response of individuals to prevalence (see e.g. [1]). As discussed by d'Onofrio & Manfredi [2], the concept of information index is an extension of the idea of the prevalence–dependent contact rate.

Consider the scenario of an epidemic outbreak that can be addressed by the public health system through campaigns aimed at raising public awareness regarding the use of protective tools (for example, vaccination, social distancing, bed–net in case of mosquito–borne diseases, etc). Assume also that the protective actions are not mandatory for the individuals (or else, they are mandatory but local authorities are unable to ensure a fully respect of the rules). Then, the final choice to use or not use the protective tools is therefore partially or fully determined by the available information on the state of the disease in the community. The information takes time to reach the population (due to time–consuming procedures such as clinical tests, notification of cases, the collecting and propagation of information and/or rumors, etc) and the population keeps the memory of the past values of the infection (like prevalence or incidence). Therefore, according to the idea of information–dependent epidemic models [3, 4], an *information index* M should be considered, which is defined in terms of a delay τ, a memory kernel K and a function $\tilde{g}$ which describes the information that is relevant to the public in determining its final choice to adopt or not to adopt the protective measure (the *message* function).

Therefore, the information index is given by the following distributed delay:

$$M(t) = \int_{-\infty}^{t} \tilde{g}\left(x_1(\tau), x_2(\tau), \ldots, x_n(\tau)\right) K(t-\tau) d\tau. \tag{S.1}$$

Here, the message function $\tilde{g}$ depends generically on the state variables, say x_1, x_2, $\ldots$, x_n, but it may specifically depend only on prevalence [5–8], incidence [9] or other relevant quantities like the vaccine side effects [10]. One may assume that:

$$\tilde{g} = \begin{cases} 0 & \text{if} \quad t < t_0 \\ g(x_1, x_2, \ldots, x_n) & \text{if} \quad t \geq t_0 \end{cases}$$

The delay kernel $K(\cdot)$ in (S.1) is a positive function such that $\int_0^{+\infty} K(t)dt = 1$. It represents the weight given to past history of the disease. The Erlangian family $Erl_{n,a}(t)$ is a good candidate for delay kernel since it may represent both an exponentially fading memory (when $n = 1$) and a memory more focused in the past (when $n > 1$). Moreover, when an Erlangian memory kernel is used, one can apply the so called 'linear chain trick' [11] to obtain a system ruled by ordinary differential equations. For example, in the case of exponentially fading memory (or *weak kernel* $Erl_{1,a}(t)$), the dynamics of the information index is ruled by

$$\dot{M} = a\left(g\left(x_1, x_2, \dots, x_n\right) - M\right).$$

For further details regarding the information index, see [3,4].

S.2 The *next generation* matrix method

Following the procedure and the notations adopted by Diekmann *et al.* [12] and Van den Driessche & Watmough [13], we derive the control reproduction number, $\mathcal{R}_C$. Let us consider the r.h.s. of equations (1b)–(1c)–(1d)–(1e)–(1f) of the main text (the balance equations for the infected compartments), and distinguish the new infections appearance from the other rates of transfer, by defining the vectors

$$\mathcal{F} = \begin{pmatrix} \beta(M)\dfrac{S}{N-Q}(\varepsilon_p I_p + \varepsilon_m I_m + \varepsilon_s I_s) \\ 0 \\ 0 \\ 0 \\ 0 \end{pmatrix}$$

and

$$\mathcal{V} = \begin{pmatrix} (\rho+\mu)E \\ -\rho E + (\eta+\mu)I_p \\ -p\eta I_p + (\gamma(M) + \sigma_m + \nu_m + \mu)I_m \\ -(1-p)\eta I_p - \sigma_m I_m - \sigma_q Q + (\nu_s + \delta + \mu)I_s \\ -\gamma(M)I_m + (\sigma_q + \nu_q + \mu)Q \end{pmatrix}.$$

The Jacobian matrices of $\mathcal{F}$ and $\mathcal{V}$ evaluated at model (1)–(2) disease–free equilibrium

$$DFE = \left(\frac{\Lambda}{\mu}, 0, 0, 0, 0, 0, 0, 0\right)$$

read, respectively,

$$F = \begin{pmatrix} 0 & (\beta_b - \beta_0)\varepsilon_p & (\beta_b - \beta_0)\varepsilon_m & (\beta_b - \beta_0)\varepsilon_s & 0 \\ 0 & 0 & 0 & 0 & 0 \\ 0 & 0 & 0 & 0 & 0 \\ 0 & 0 & 0 & 0 & 0 \\ 0 & 0 & 0 & 0 & 0 \end{pmatrix}$$

and

$$V = \begin{pmatrix} \rho+\mu & 0 & 0 & 0 & 0 \\ -\rho & \eta+\mu & 0 & 0 & 0 \\ 0 & -p\eta & \gamma_0 + \sigma_m + \nu_m + \mu & 0 & 0 \\ 0 & -(1-p)\eta & -\sigma_m & \nu_s + \delta + \mu & -\sigma_q \\ 0 & 0 & -\gamma_0 & 0 & \sigma_q + \nu_q + \mu \end{pmatrix}.$$

The control reproduction number is given by the spectral radius of the *next generation* matrix FV^{-1} [12, 13]. It is easy to check that FV^{-1} has positive elements on the first row, being the other ones null. Thus, $\mathcal{R}_C = (FV^{-1})_{11}$, yielding

$$\mathcal{R}_C = (\beta_b - \beta_0)\rho \left[\frac{\varepsilon_p}{B_1 B_2} + \frac{\varepsilon_m p \eta}{B_1 B_2 B_4} + \frac{\varepsilon_s (1-p)\eta}{B_1 B_2 B_6} + \frac{\varepsilon_s p \eta \sigma_m}{B_1 B_2 B_4 B_6} + \frac{\varepsilon_s p \eta \gamma_0 \sigma_q}{B_1 B_2 B_4 B_5 B_6} \right], \tag{S.2}$$

with $B_1 = \rho + \mu$, $B_2 = \eta + \mu$, $B_4 = \gamma_0 + \sigma_m + \nu_m + \mu$, $B_5 = \sigma_q + \nu_q + \mu$, $B_6 = \nu_s + \delta + \mu$. Similarly one can prove that the basic reproduction number is given by

$$\mathcal{R}_0 = \beta_b \rho \left[\frac{\varepsilon_p}{B_1 B_2} + \frac{\varepsilon_m p \eta}{B_1 B_2 B_3} + \frac{\varepsilon_s (1-p)\eta}{B_1 B_2 B_6} + \frac{\varepsilon_s p \eta \sigma_m}{B_1 B_2 B_3 B_6} \right], \tag{S.3}$$

with $B_3 = \sigma_m + \nu_m + \mu = B_4 - \gamma_0$. The first two terms in the r.h.s of (S.2) describe the contributions of post–latent infectious and asymptomatic/mildly symptomatic infectious, respectively, to the production of new infections close to the disease–free equilibrium. The third, the fourth and the fifth term in (S.2) represent the contribution of infectious with severe symptoms. The severe symptoms can onset soon after the post–latent phase (third term) or after a mildly symptomatic phase (fourth term). In case of quarantine, severe symptoms can onset also during such a stage (fifth term). Note that the last term is missing in the basic reproduction number (S.3), where the possibility for people to be quarantined is excluded. Note also that $\mathcal{R}_C = \mathcal{R}_0$ when $\beta_0 = \gamma_0 = 0$.

S.3 Epidemiological parameters

As done by Gumel *et al.* [14], we adopt an SEIR–like model with demography and constant net inflow of susceptibles Λ. Including a net inflow of susceptible individuals into the model allows to consider not only new births (which can be assumed to be approximately constant due to the short time span of our analysis), but also immigration, which played a role during the lockdown in Italy. Therefore, the net inflow of susceptibles is given by

$$\Lambda = b\bar{N} + \Lambda_0. \tag{S.4}$$

In (S.4), the parameter Λ_0 is the inflow term due to immigration and $b\bar{N}$ is the inflow term due to births, where b is the birth rate and $\bar{N}$ denotes the total population at the beginning of the epidemic.

The most recent data by the Italian National Institute of Statistics [15] refer to January 1, 2019 and provide a country–level birth rate $b = 7.2/1,000$ years^{-1} and a natural death rate $\mu = 10.7/1,000$ years^{-1}, as well as a resident population of about

$$\bar{N} \approx 60.360 \cdot 10^6 \tag{S.5}$$

inhabitants. Fluctuations in a time window of just over a year are considered negligible.
Since global travel restrictions were implemented during the COVID–19 epidemic outbreak [16], we assume that the immigration inflow term Λ_0 accounts only of repatriation of citizens to their countries of origin (Italy in that case) due to the COVID–19 pandemic [17]. In all airports, train stations, ports and land borders travellers' health conditions have been tested via thermal scanners. Although the effectiveness of such screening method is largely debated [18], for the sake of simplicity, we assume that the inflow enters only the susceptible compartment. On the basis of data communicated by the Italian Ministry of Foreign Affairs and International Cooperation [19], a reasonable value for Λ_0 seems to be $\Lambda_0 = 4,000/7$ days^{-1},

namely the average number of repatriated citizens was 4,000 *per* week. Being $\Lambda = b\bar{N} + \Lambda_0$, we finally obtain $\Lambda \approx 1.762 \cdot 10^3$ days^{-1}.

Epidemiological data are based on the current estimates disseminated by national and international health organizations [20–25] or inferred by modelling studies [26–28]. More precisely, the median incubation period is estimated to be from 5–6 days, with a range from 1–14 days, and identification of the virus in respiratory tract specimens occurs 1–2 days before the onset of symptoms [21, 22]. Hence, we set the latency (ρ) and post–latency (η) rates to 1/5.25 days^{-1} and 1/1.25 days^{-1}, respectively.
From [26], the specific baseline transmission rates for the post–latent ($\varepsilon_p\beta_b$), asymptomatic/mildly symptomatic ($\varepsilon_m\beta_b$) and severely symptomatic ($\varepsilon_s\beta_b$) cases are such that $\varepsilon_m/\varepsilon_p = 0.033$ and $\varepsilon_s/\varepsilon_m = 1.03$. They are in accordance with the observation of high viral load close to symptoms onset (suggesting that SARS–CoV–2 can be easily transmissible at an early stage of infection), and with the absence of reported significant difference in viral load in asymptomatic and symptomatic patients [22]. We set $\beta_b = 2.25$ days^{-1}, which, together with the other parameters, leads to the basic reproduction number $\mathcal{R}_0 \approx 3.49$, a value falling within the ranges estimated in several sources [21, 22, 25, 26].

As made by Kantner & Koprucki [27], we consider that just 8% of infectious individuals shows serious symptoms immediately after the incubation phase, yielding $p = 0.92$. Nonetheless, people with initial mild symptoms may become seriously ill and develop breathing difficulties, requiring hospitalization. It is estimated that about 1 in 5 people with COVID–19 shows a worsening of symptoms [23] within 4–5 days from onset [20], giving $\sigma_m = 0.2/4.5 \approx 0.044$ days^{-1}. Instead, the possibility that the aggravation happens during the quarantine period is assumed to be more rare: $\sigma_q = 0.001$ days^{-1}.

Governmental efforts in identifying and quarantining positive cases have been implemented since the early stage of epidemics (at February 24, 94 quarantined people were already registered [29]), hence we consider the daily mandatory quarantine rate of asymptomatic/mildly symptomatic individuals (γ_0) for the whole time horizon. From current available data, it seems hard to catch an uniform value for γ_0 because it largely depends on the sampling effort, namely the number of specimen collections (swabs) from persons under investigation, that varied considerably across Italian regions and in the different phases of the outbreak [20, 29]. Since our model does not account for such territorial peculiarities and in order to reduce the number of parameters to be estimated, we assume that $\gamma_0 = 1.3\sigma_q$, namely it is 30% higher than the daily rate at which members of the I_m class hospitalize, yielding $\gamma_0 \approx 0.057$ days^{-1}.

Following the approach adopted by Gumel *et al.* [14] for a SARS–CoV epidemic model, based on the formula given by Day [30], we estimate the disease–induced death rate as

$$\delta = (1 - \mu T)\frac{X}{T},$$

where X is the case fatality and T is the expected time from hospitalization until death. From [20], we approximate $X = 13\%$ and $T = 6$ days (it is 9 days for patients that were transferred to intensive care and 5 days for those were not), yielding $\delta \approx 0.022$ days^{-1}. Similarly, the recovery rates ν_j with $j \in \{m, q, s\}$ are estimated as

$$\nu_j = (1 - \mu T_j)\frac{1 - X}{T_j},$$

where T_j is the expected time until recovery or expected time in quarantine/hospitalization. Preliminary data indicate that the virus can persist for up to eight days from the first detection in moderate cases and for longer periods in more severe cases [22], suggesting $T_m = 6$ days is an appropriate value. As far as the time spent in hospitalization or quarantine is concerned, in the lack of exact data we assume $T_s < T_q$ because hospitalized individuals are likely to receive a partly effective, experimental treatment: mainly antibiotics, antivirals and corticosteroids [20]. Moreover, shortages in hospital beds and intensive care

units (ICUs) led to as prompt as possible discharge [31]. In particular, we set $T_s = 18$ and $T_q = 25$ days, by accounting also for prolonged quarantine time due to delays in test response (if any) and for WHO recommendations of an additional two weeks in home isolation even after symptoms resolve [24].
Crucially, we also estimate the initial exponential rate of case increase (say, g_0), by computing the dominant eigenvalue of the system's Jacobian matrix, evaluated at the disease–free equilibrium. It provides $g_0 \approx 0.247$ days^{-1}, in accordance to that given by Gatto *et al.* [26].

S.4 Initial conditions

In order to provide appropriate initial conditions, we consider the official national data at February 24, 2020 archived on [29]. In particular, we take the number of mandatorily quarantined individuals (at that time, they coincide with Q being the voluntary component negligible) and the hospitalized people (I_s). Then, we use system (1)–(2) of the main text to simulate the temporal evolution of the epidemics prior to February 24, by imposing an initial condition of one exposed case Δt_0 days before in a population of $\bar{N}$ individuals, with $\bar{N}$ given in (S.5). We assume $\beta_0 = 0$ and γ_0 as in Table 1 of the main text (no social distance restrictions were initially implemented, but quarantine efforts were active since then) and disregard the effect of information on the human social behaviors in this phase ($\alpha = D = 0$ in (1)–(2) of the main text). The length of temporal interval Δt_0 is tuned in order to reproduce the official values released for Q and I_s at February 24 and provide estimations for the other state variables, as reported in Table 1 of the main text. We obtain $\Delta t_0 = 31.9$, indicating that the virus circulated since the end of January, as predicted also by Gatto *et al.* [26] and Giordano *et al.* [32].

S.5 Mitigation measures enacted by the Italian government

Italy was the first European country affected by COVID–19. After the first officially confirmed case (the so–called 'patient one') on February 21, 2020 in the Lodi province, several suspected cases emerged in the south and southwest of the Lombardy region. A 'red zone' encompassing 11 municipalities was instituted on February 22 and put on lockdown to contain the emerging threat. On March 8, the red zone was extended to the entire Lombardy region and 14 more northern Italian provinces, while the rest of Italy implemented social distancing measures. A leak of a draft of this decree prompted a panic reaction with massive movement of people towards Italian regions, especially from the north to the south [33]. The next day, a decree evocatively entitled 'I'm staying at home' was signed: the lockdown was declared for the whole country with severe limitations to mobility and other progressively stricter restrictions.

Soon after, on March 11, 2020, the lockdown was enforced with all commercial and retail businesses – except those providing essential services – closed down [34]. Finally, on March 22, 2020, the *phase one* of restrictions was completed when a *full* lockdown was imposed by closing all non essential companies and industrial plants [35]. On May 4, Italy entered the *phase two*, representing the starting point of a gradual relaxation of the restriction measures. One week later, shops also reopened and the restrictions on mobility were essentially eliminated, with the only obligation in many regions to use protective masks [36].

References

[1] Capasso V, Serio G. A generalization of the Kermack–McKendrick deterministic epidemic model. Mathematical Biosciences. 1978;42(1):43 – 61. Available from: http://www.sciencedirect.com/science/article/pii/0025556478900068.

[2] d'Onofrio A, Manfredi P. The interplay between voluntary vaccination and reduction of risky behavior: a general behavior–implicit SIR model for vaccine preventable infections. In: Current Trends in Dynamical Systems in Biology and Natural Sciences. Cham, CH: Springer; 2020. p. 185–203.

[3] Manfredi P, d'Onofrio A, editors. Modeling the Interplay Between Human Behavior and the Spread of Infectious Diseases. New York: Springer; 2013.

[4] Wang Z, Bauch CT, Bhattacharyya S, d'Onofrio A, Manfredi P, Perc M, et al. Statistical physics of vaccination. Physics Reports. 2016;664:1–113.

[5] Buonomo B, Della Marca R. Oscillations and hysteresis in an epidemic model with information–dependent imperfect vaccination. Mathematics and Computers in Simulation. 2019;162:97 – 114. Available from: http://www.sciencedirect.com/science/article/pii/S0378475419300242.

[6] d'Onofrio A, Manfredi P. Information–related changes in contact patterns may trigger oscillations in the endemic prevalence of infectious diseases. Journal of Theoretical Biology. 2009;256(3):473 – 478. Available from: http://www.sciencedirect.com/science/article/pii/S0022519308005377.

[7] d'Onofrio A, Manfredi P, Salinelli E. Vaccinating behaviour, information, and the dynamics of SIR vaccine preventable diseases. Theoretical Population Biology. 2007;71(3):301 – 317. Available from: http://www.sciencedirect.com/science/article/pii/S0040580907000020.

[8] d'Onofrio A, Manfredi P, Salinelli E. Fatal SIR diseases and rational exemption to vaccination. Mathematical Medicine and Biology: a Journal of the IMA. 2008;25(4):337–357.

[9] Buonomo B, d'Onofrio A, Lacitignola D. Modeling of pseudo–rational exemption to vaccination for SEIR diseases. Journal of Mathematical Analysis and Applications. 2013;404(2):385–398.

[10] d'Onofrio A, Manfredi P, Salinelli E. Vaccinating behaviour and the dynamics of vaccine preventable infections. In: Modeling the Interplay Between Human Behavior and the Spread of Infectious Diseases. New York: Springer; 2013. p. 267–287.

[11] MacDonald N. Biological delay systems: linear stability theory. Cambridge: Cambridge University Press; 2008.

[12] Diekmann O, Heesterbeek JAP, Metz JAJ. On the definition and the computation of the basic reproduction ratio R_0 in models for infectious diseases in heterogeneous populations. Journal of Mathematical Biology. 1990;28(4):365–382. Available from: https://doi.org/10.1007/BF00178324.

[13] Van den Driessche P, Watmough J. Reproduction numbers and sub–threshold endemic equilibria for compartmental models of disease transmission. Mathematical Biosciences. 2002;180(1):29–48.

[14] Gumel AB, Ruan S, Day T, Watmough J, Brauer F, Van den Driessche P, et al. Modelling strategies for controlling SARS outbreaks. Proceedings of the Royal Society of London Series B: Biological Sciences. 2004;271(1554):2223–2232.

[15] ISTAT, Istituto Nazionale di Statistica. Demography in Figures; 2020. (Accessed May 13, 2020). http://www.demo.istat.it/index.html.

[16] UNWTO, World Tourism Organization. COVID–19 related travel restrictions. A global review for tourism. Second report as of 28 April 2020; 2020. (Accessed May 5, 2020). https://webunwto.s3.eu-west-1.amazonaws.com/s3fs-public/2020-04/TravelRestrictions%20-%2028%20April.pdf.

[17] EPRS, European Parliamentary Research Service. Repatriation of EU citizens during the COVID–19 crisis; 2020. (Accessed May 5, 2020). https://www1.europarl.europa.eu/RegData/etudes/BRIE/2020/649359/EPRS_BRI(2020)649359_EN.pdf.

[18] Quilty BJ, Clifford S, CMMID nCoV working group2, Flasche S, Eggo RM. Effectiveness of airport screening at detecting travellers infected with novel coronavirus (2019–nCoV). Eurosurveillance. 2020;25(5):2000080.

[19] Italian Ministry of Foreign Affairs and International Cooperation. Foreign Ministry commitment for Italians abroad; 2020. (Accessed May 5, 2020). https://www.esteri.it/mae/en/sala_stampa/archivionotizie/approfondimenti/impegno-della-farnesina-per-gli-italiani-all-estero.html.

[20] ISS, Istituto Superiore di Sanità, EpiCentro. SARS-CoV-2 – Latest available data; 2020. (Accessed May 10, 2020). https://www.epicentro.iss.it/en/coronavirus/.

[21] WHO, World Health Organization. Coronavirus disease (COVID–19) Pandemic; 2020. (Accessed May 18, 2020). https://www.who.int/emergencies/diseases/novel-coronavirus-2019.

[22] ECDC, European Centre for Disease Prevention and Control. Disease background of COVID–19; 2020. (Accessed May 2, 2020). https://www.ecdc.europa.eu/en/2019-ncov-background-disease.

[23] Italian Ministry of Health. FAQ - Covid–19, questions and answers; 2020. (Accessed May 4, 2020). http://www.salute.gov.it/portale/nuovocoronavirus/dettaglioFaqNuovoCoronavirus.jsp?lingua=english&id=230#2.

[24] WHO, World Health Organization. Home care for patients with COVID–19 presenting with mild symptoms and management of their contacts; 2020. (Accessed May 18, 2020). https://www.who.int/publications-detail/home-care-for-patients-with-suspected-novel-coronavirus-(ncov)-infection-presenting-with-mild-symptoms-and-management-of-contacts.

[25] Flaxman S, Mishra S, Gandy A, Unwin HJT, Mellan TA, Coupland H, et al. Estimating the effects of non–pharmaceutical interventions on COVID–19 in Europe. Nature. 2020;584:257–261.

[26] Gatto M, Bertuzzo E, Mari L, Miccoli S, Carraro L, Casagrandi R, et al. Spread and dynamics of the COVID–19 epidemic in Italy: Effects of emergency containment measures. Proceedings of the National Academy of Sciences. 2020;117(19):10484–10491. Available from: https://www.pnas.org/content/early/2020/04/22/2004978117.

[27] Kantner M, Koprucki T. Beyond just "flattening the curve": Optimal control of epidemics with purely non–pharmaceutical interventions. Journal of Mathematics in Industry. 2020;10(23).

[28] Di Domenico L, Pullano G, Sabbatini CE, Boëlle PY, Colizza V. Impact of lockdown on COVID–19 epidemic in Île–de–France and possible exit strategies. BMC Medicine. 2020;18(240).

[29] Italian Ministry of Health. Dati COVID–19 Italia; 2020. (Accessed August 15, 2020). https://github.com/pcm-dpc/COVID-19.

[30] Day T. On the evolution of virulence and the relationship between various measures of mortality. Proceedings of the Royal Society of London Series B: Biological Sciences. 2002;269(1498):1317–1323.

[31] ESA, European Society of Anaesthesiology. Dynamics of ICU patients and deaths in Italy and Lombardy due to Covid–19. Analysis updated to 30–March, Day #38 evening; 2020. (Accessed May 15, 2020). https://www.esahq.org/esa-news/dynamics-of-icu-patients-and-deaths-in-italy-and-lombardy-due-to-covid-19-analysis-updated-to-30-march-day-38-evening.

[32] Cereda D, Tirani M, Rovida F, Demicheli V, Ajelli M, Poletti P, et al. The early phase of the COVID–19 outbreak in Lombardy, Italy. arXiv. 2020.

[33] The Guardian. Leaked coronavirus plan to quarantine 16m sparks chaos in Italy; 2020. (Accessed May 15, 2020). https://www.theguardian.com/world/2020/mar/08/leaked-coronavirus-plan-to-quarantine-16m-sparks-chaos-in-italy.

[34] Italian Civil Protection Department. Chronology of main steps and legal acts taken by the Italian Government for the containment of the COVID–19 epidemiological emergency; 2020. (Accessed May 11, 2020). http://www.protezionecivile.gov.it/documents/20182/1227694/Summary+of+measures+taken+against+the+spread+of+C-19/c16459ad-4e52-4e90-90f3-c6a2b30c17eb.

[35] ANSA, Agenzia Nazionale Stampa Associata. Coronavirus: Conte tightens lockdown, closes all non–essential businesses, after almost 800 deaths in 24 hours; 2020. (Accessed May 5, 2020). https://www.ansa.it/english/news/2020/03/20/stay-home-or-well-take-other-tough-measures-di-maio_fe91986d-3a31-4dac-8671-44feab0bd506.html.

[36] Italian Ministry of Health. Covid–19. Phase two: what's opening and what you can do; 2020. (Accessed May 11, 2020). http://www.salute.gov.it/portale/nuovocoronavirus/dettaglioNotizieNuovoCoronavirus.jsp?lingua=english&id=4670.

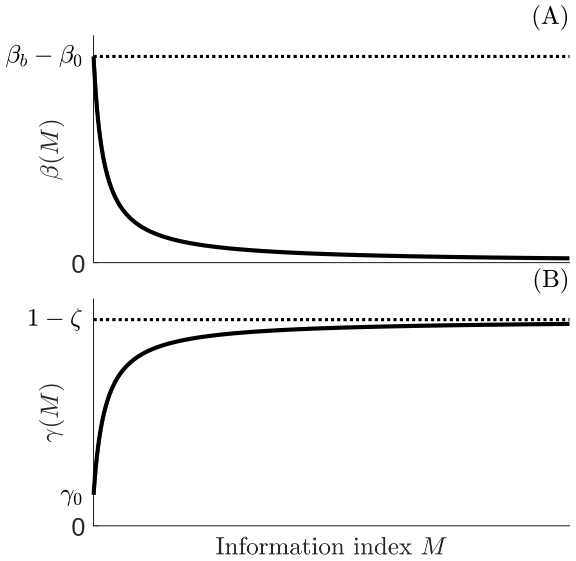

Figure S.1: Representative shapes of the transmission rate (panel A) and the quarantine rate (panel B) as functions of the information index M.

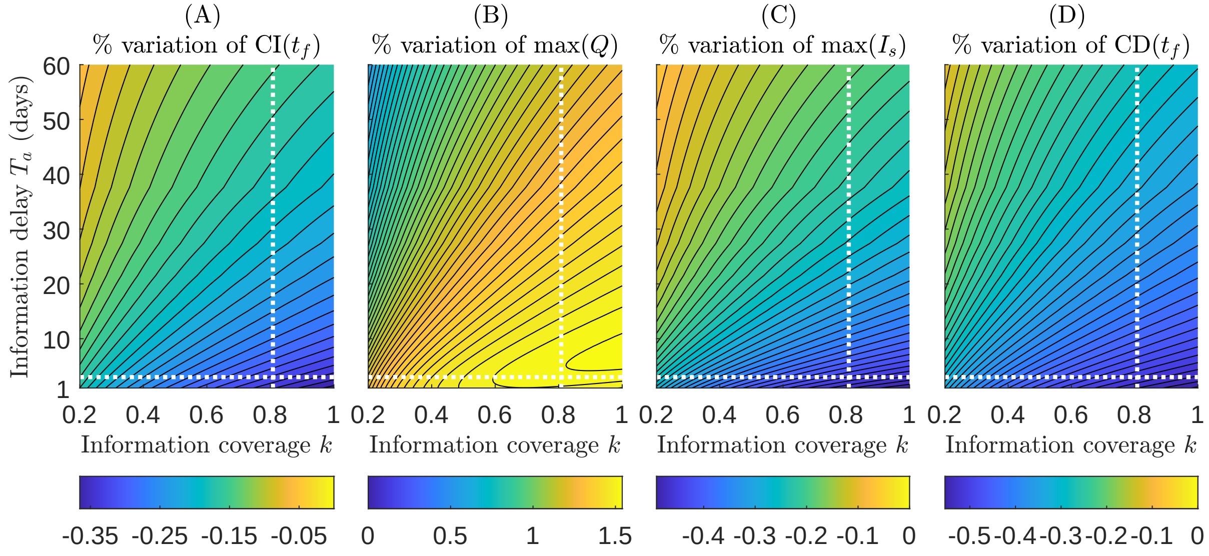

Figure S.2: Contour plots of the percentage relative change w.r.t the unresponsive case $\alpha = D = 0$ versus information coverage k and average delay $T_a = a^{-1}$. Panel A: cumulative incidence CI(t_f) evaluated at the last day of the considered time frame, i.e. $t_f = 85$, corresponding to May 18, 2020. Panel B: peak of quarantined individuals. Panel C: peak of hospitalized individuals. Panel D: final cumulative deaths CD(t_f). The intersection between dotted white lines indicates the values corresponding to the baseline scenario $k = 0.8$, $T_a = 3$ days. Other parameter values are given in Tables 1 and 2 of the main text.
